# Circulating tumour cells and hypercoagulability: a lethal relationship in metastatic breast cancer

**DOI:** 10.1007/s12094-019-02197-6

**Published:** 2019-08-31

**Authors:** C. C. Kirwan, T. Descamps, J. Castle

**Affiliations:** 1 Division of Cancer Sciences, Faculty of Biology, Medicine and Health, School of Medical Sciences, Manchester Cancer Research Centre, University of Manchester, Wilmslow Road, Manchester, M20 4GJ UK; 2 Wythenshawe Hospital, Manchester University NHS Foundation Trust, Manchester Academic Health Science Centre, Southmoor Road, Wythenshawe, Manchester, M23 9LT UK; 3 Centre for Cancer Biomarker Sciences, Cancer Research UK Manchester Institute, University of Manchester, Alderley Park, Macclesfield, SK10 4TG UK

**Keywords:** Coagulation, CTC, TAT, Fibrinogen, d-Dimer, Survival

## Abstract

**Purpose:**

Circulating tumour cells (CTCs) are a marker of poor prognosis and are associated with increased risk of venous thromboembolism in metastatic breast cancer (MBC). We aimed to determine if the presence of CTCs and plasma markers of hypercoagulability [thrombin–antithrombin III (TAT), fibrinogen and d-dimer] are biomarkers of survival in MBC.

**Methods/patients:**

In a prospective study of MBC patients, CTC (CellSearch^®^) enumeration and plasma TAT, fibrinogen and d-dimer measured prior to commencement of treatment for disease progression were correlated to overall survival.

**Results:**

At study completion, of 50 MBC patients recruited (median age 59 years, range 36–82), 40 patients had died (median survival 417 days, range 58–2141). CTCs (≥ 1/7.5 ml) were identified in 16 patients (median number of cells per 7.5 ml, 3 (range 1–31) and were associated with systemic hypercoagulability (medians TAT: 8.1 vs. 5.2 ng/ml, *p* = 0.03; fibrinogen: 4.3 vs. 3.1 g/l, *p* = 0.03; d-dimer: 1327 vs. 683 ng/ml, *p* = 0.0001). At 1 year, of 16 patients with ≥ 1 CTC, 7 had died (44%), compared to 5 of 26 (19%) patients in the no-CTC group. The presence of ≥ 1 CTC was associated with a trend for reduced overall survival (median 455 days vs. 614 days, *p* = 0.15). Plasma TAT inversely correlated with survival and was significantly higher in patients dying within 1 year (median 9.8 vs. 5.2 ng/ml, *p* = 0.004) whilst d-dimer showed a trend for reduced 1-year survival (median 1211 vs. 817 ng/ml, *p* = 0.06). MBC patients with combined high d-dimer (≥ 895 ng/ml) and CTC positivity (≥ 1/7.5 ml whole peripheral blood) had significantly reduced survival (*p* = 0.04).

**Conclusions:**

The correlation between CTCs, hypercoagulability and reduced survival in MBC suggests the coagulation system supports tumour cell metastasis and is, therefore, a potential therapeutic target.

**Electronic supplementary material:**

The online version of this article (10.1007/s12094-019-02197-6) contains supplementary material, which is available to authorized users.

## Introduction

Venous thromboembolism (VTE) in cancer is common and a major cause of death. Breast cancer is associated with a three- to fourfold increased risk of venous thromboembolism (VTE) compared to age-matched women without cancer [1, 2]. Cancer patients who develop VTE have a worse cancer prognosis than those that remain free of VTE, implying VTE is a surrogate marker for aggressive cancer [3–5].

Approximately half of all cancer patients and 90% of metastatic patients exhibit coagulation abnormalities. This is reflected in activation of the clotting cascade [6], platelet activation [7] and alterations of fibrinolytic activity [8] resulting in clinical and biochemical evidence of coagulation [9, 10]. Thrombin–antithrombin III (TAT) complex plasma concentration is used to assess activation of the coagulation system as a surrogate marker for activated thrombin [11]. Plasma TAT and d-dimer are higher in early-breast cancer patients than in healthy controls [12]. Circulating fibrinogen, which is converted to fibrin by thrombin, has showed utility in distinguishing breast cancer from benign breast disease [13], and increases with disease progression [14]. d-dimer (a product of fibrin degradation and the end product of coagulation activation) is increased in breast cancer, with raised levels being found in 58% of patients with involved lymph nodes, and only 8% of patients without lymph node disease [10].

Many coagulation factors can be identified in the tumour stroma and cancer cells [15]. Tumour cells can activate all components of the haemostatic system, for example the procoagulant potential of cancer cells has a linear relationship with their tissue factor expression [16]. However, there appears to be a two-way association between coagulation and cancer. Components of the haemostatic system promote tumour growth and metastasis, with thrombin increasing proliferation, migration and angiogenesis in preclinical models [17, 18]. Tissue factor has furthermore been identified as a promoter of cancer stem cell activity [19].

Circulating tumour cells (CTCs) are cells from a primary tumour that have entered the vasculature or lymphatics, and are the principal mechanism for development of metastases. First described by Ashworth [20], the field of CTC research has grown exponentially, with the hope that CTC assessment will act as real-time predictive and prognostic biomarker. Using the commercially available CellSearch^®^ assay (Menarini Group, Italy), CTC number predicts for reduced progression-free survival (PFS) and overall survival (OS) in several MBC studies [21–24]. CellSearch CTC number is also a potential prognostic biomarker and a predictive biomarker for response to chemotherapy and endocrine therapy in MBC patients [25, 26].

## Hypothesis

We hypothesise that the symbiotic relationship between CTCs and plasma coagulation results in more effective dissemination of metastases, reflects more aggressive disease and correlates with reduced survival in MBC. We aimed to determine if the combination of CTC enumeration and quantification of systemic hypercoagulability [thrombin–antithrombin III (TAT), fibrinogen and d-dimer] is a biomarker of reduced overall survival in MBC.

## Methods

A prospective cohort of metastatic breast cancer patients (*n* = 50) were recruited at The Christie NHS Foundation Trust Hospital (Manchester, UK) between February 2013 and June 2015 (TuFClot, Tumour Fragments and Clotting). Follow-up was until July 2019. All had either a new diagnosis of metastatic disease or new evidence of clinical or radiological disease progression. Patients on anticoagulation therapy or with a known non-breast cancer within the last 5 years were excluded. This study was approved by UK National Research Ethics Service (NRES) Committee North West-Greater Manchester Central Ethics Committee (Ref: 12/NW/0447) and sponsored by Manchester University NHS Foundation Trust. Written informed consent was obtained from all individual participants included in the study.

This clinical study report has been written with the guidance of the reporting recommendations for tumour marker prognostic studies (REMARK) [27].

### Blood sampling

Peripheral venous blood was collected using CellSave preservative (Menarini, Italy, Cat# 7900005) and citrate vacutainer tubes (BD Biosciences, NJ USA, Cat# 363095) for CellSearch^®^ circulating tumour cell (CTC) analysis and platelet poor plasma preparation, respectively. Blood was collected prior to treatment for disease progression. CellSave whole blood samples were stored at room temperature until analysis.

For TAT, fibrinogen and d-dimer analysis, citrate tubes were centrifuged within 60 min of blood taking in a pre-cooled centrifuge at 4 °C for 20 min at 2500 *g*. Plasma was pipetted into a clear tube leaving the cell pellet and centrifuged again at 4 °C for 20 min at 2500 *g*. The platelet poor plasma was aliquoted into cryovials and stored at − 80 °C until analysis, with the final 0.5 ml in the clear tube discarded.

Analysis of whole blood and plasma samples was carried out by analysts trained in good clinical practice (GCP) who were blinded to patient outcome data.

### CellSearch^®^ circulating tumour cell (CTC) analysis

CellSave blood samples (7.5 ml) were processed by the CellSearch^®^ system in the Clinical and Experimental Pharmacology (CEP) group at the Cancer Research UK Manchester Institute (CRUK MI) as described elsewhere [21]. Briefly, CTCs are immunomagnetically separated from other blood components by EpCAM (epithelial cell adhesion molecule) antibody-conjugated beads and then stained for cytokeratins (CKs 8, 18 and 19) and CD45 in a fluorescent-based approach. CTCs are defined as CK+ CD45− cells over 4 µm in diameter (Supp. Figure 1). CellSearch CTC Control Kits (Cat# 7900003) were used as a quality control to ensure reagent, instrument and operator performance.

Supplementary Figure 1 represents the gallery of images shown on the CellSearch^®^ Analyzer after CTC enrichment from blood using EpCAM antibodies and staining. Cells ≥ 4 µm immunofluorescently staining for cytokeratins and not CD45 are scored as CTCs. The event shown was scored as a CTC by trained analysts.

### TAT, fibrinogen and d-dimer analysis

Plasma was analysed using Siemens AG (Berlin, Germany) Enzygnost^®^ TAT micro-enzyme immunoassays (Cat# OWMG15) in the CEP group at CRUK MI. Validation work was carried out prior to analysis to ensure fit for purpose. Plasma was analysed for fibrinogen and d-dimer using a Werfen (Warrington, UK) ACL TOP 500 automated haemostasis testing system in the Haematology Department at Wythenshawe Hospital. HemosIL^®^ Q.F.A. Thrombin clotting assay (Cat# 0020301700) and d-dimer HS 500 immunoassay (Cat# 0020500100) reagents were used to quantify fibrinogen and d-dimer, respectively.

### Statistical analysis

GraphPad Prism software version 7 (CA, USA) was used for primary statistical analysis and graph preparation. Raw TAT, fibrinogen and d-dimer data was Log_2_ transformed to allow parametric testing after 1 was added to each raw value to prevent Log_2_ values ≤ 0. Normality of the transformed coagulation marker data was confirmed by D’Agostino and Pearson testing. Coagulation marker groups were compared using unpaired two-tailed *t* tests, and raw coagulation values were dichotomised around the median value to allow comparison of high versus low.

The percentage survival at 1 year was reported. Overall survival was measured as days from study entry till death or censored at last follow-up (with a minimal follow-up of 365 days), until July 2019. Log-rank (Mantel–Cox) regression was performed to assess survival between patient groups. The proportionality of hazard assumptions for use of the Cox model was verified based on Schoenfeld residuals using the statistical computing software R [28].

## Results

Of the 50 MBC patients recruited, median age was 59 years (range 36–82), 66% were oestrogen receptor (ER) positive, 32% were Her2 positive and 12% had bone metastases only (Table 1). At 1-year follow-up 16 patients had died. At final follow-up in July 2019, 40 patients had died, median survival 417 days, range 58–2141 days.

**Table 1:** TuFClot study metastatic breast cancer patient demographics

	*n* (%)
Receptor status	
ER+, Her2−	26 (52)
ER+, Her2+	7 (14)
ER−, Her2+	9 (18)
Triple negative	8 (16)
Metastatic sites	
Bone only	6 (12)
Locally advanced	3 (6)
Single visceral site	
Liver	12 (24)
Lung	12 (24)
Brain	2 (4)
Other	1 (2)
Multiple visceral sites (≥ 2)	14 (28)

Oestrogen receptor (ER) values were reported as a Modified Quick Score (QS) for all patients. A QS value of 0–2 was considered negative, 3–8 positive

Eight patients had no CTC result due to insufficient blood sample or CellSearch equipment failure. Insufficient plasma samples led to missing patient results for TAT analysis (9 out of 50 patients), fibrinogen (16/50) and d-dimer (10/50).

The datasets generated and analysed during the TuFClot study are available in the Mendeley Data repository, https://dx.doi.org/10.17632/742ftgfg54.2 [29].

**Fig. 1:**
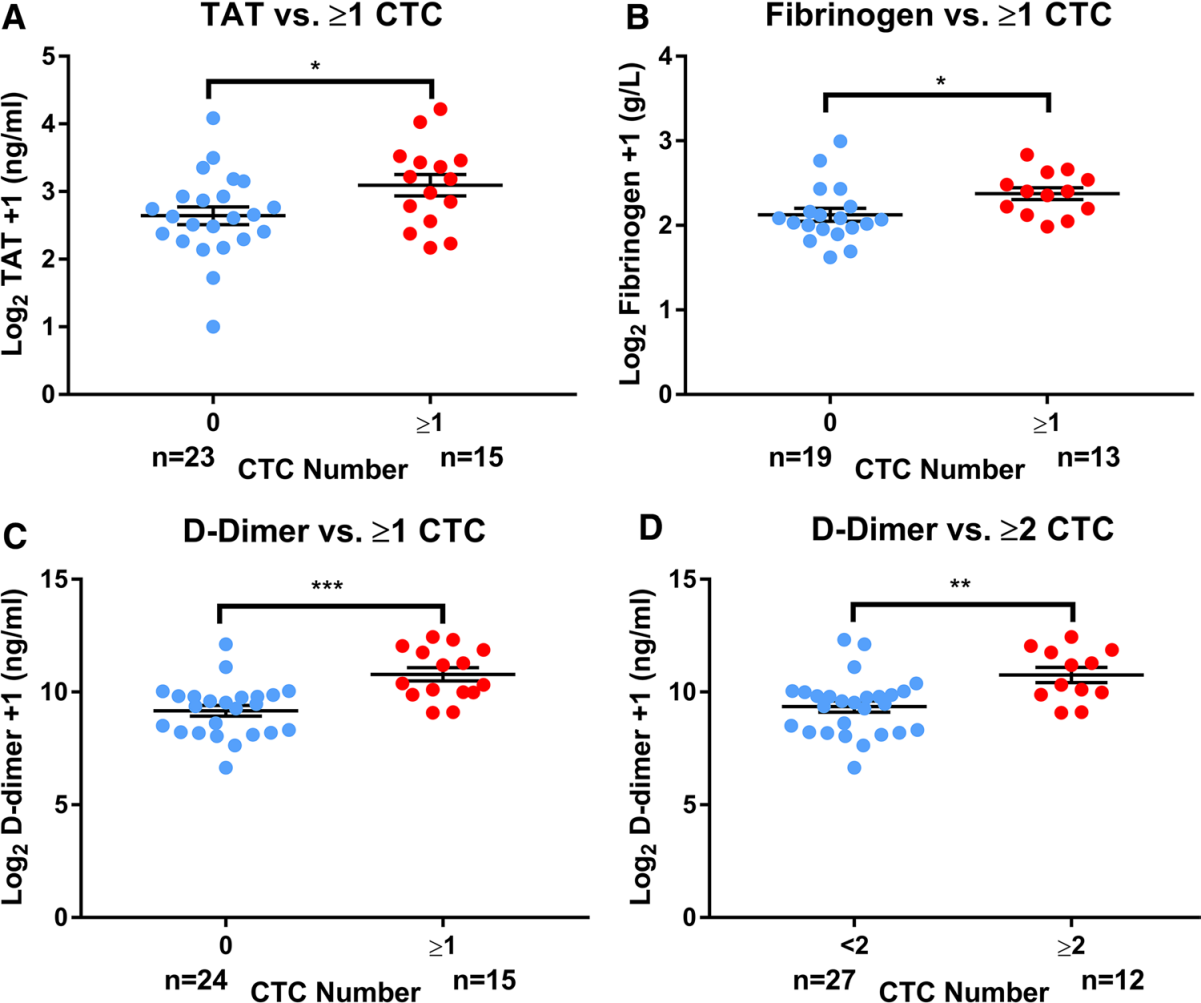
Circulating tumour cells (CTCs) are associated with increased coagulation plasma markers in metastatic breast cancer. CTC number in 7.5 ml peripheral whole blood samples enumerated by the CellSearch technology compared to plasma coagulation markers **a** thrombin–antithrombin III complex (TAT), **b** fibrinogen and **c**, **d**d-dimer in metastatic breast cancer (MBC) patients. Unpaired two-tailed *t* tests comparing Log_2_ transformed raw coagulation marker concentrations + 1 to CTC group, *p < 0.05, **p < 0.01, ***p < 0.001. Error bars show standard error of the mean (SEM); *n* number of MBC patients in each group, *TAT* thrombin–antithrombin III complex, *CTC* circulating tumour cell

Circulating tumour cells, CTCs (≥ 1/7.5 ml whole peripheral blood) were identified in 16 out of 42 MBC patients (38.1%) [median number of cells per 7.5 ml, 3 (range 1–31)]. CTC positivity (≥ 1/7.5 ml) was associated with hypercoagulability (TAT: median 8.1 vs. 5.2 ng/ml, *p* = 0.03; fibrinogen: median 4.3 vs. 3.1 g/l, *p* = 0.03; d-dimer: median 1327 vs. 683 ng/ml, *p* = 0.0001). Taking the more rigorous definition of CTC positivity as ≥ 2/7.5 ml whole peripheral blood, CTC ≥ 2/7.5 ml (*n* = 12) was also associated with increased d-dimer but not fibrinogen or TAT: median 1815 vs. 741 ng/ml, *p* < 0.01 (Fig. 1).

**Fig. 2:**
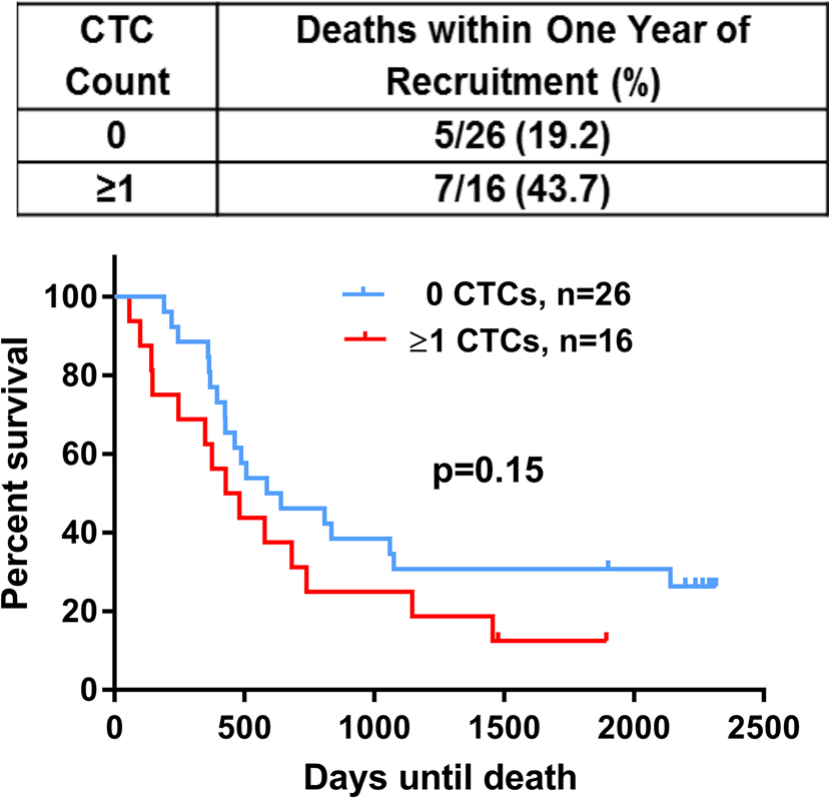
Trend for reduced overall survival in circulating tumour cell (CTC)-positive metastatic breast cancer patients. CTC number in 7.5 ml peripheral whole blood samples enumerated by the CellSearch technology was enumerated in metastatic breast cancer (MBC) patients. Log-rank (Mantel–Cox) testing compared survival in days from study entry in CTC-positive compared to CTC-negative patients. CTC positive defined as ≥ 1/7.5 ml. Assumption of proportionality was verified based on Schoenfeld residuals; *n* number of MBC patients in each group, *CTC* circulating tumour cell

CTC count did not correlate with ER or Her2 status, or the presence of visceral and locally advanced compared to bone-only metastases. The presence of ≥ 1 CTC was associated with a trend for reduced overall survival (median 455 days vs. 614 days, *p* = 0.15, Fig. 2). At 1 year, of 16 patients with ≥ 1 CTC, 7 had died (44%), compared to 5 of 26 (19%) in the no-CTC group, however, this was not statistically significant.

**Fig. 3:**
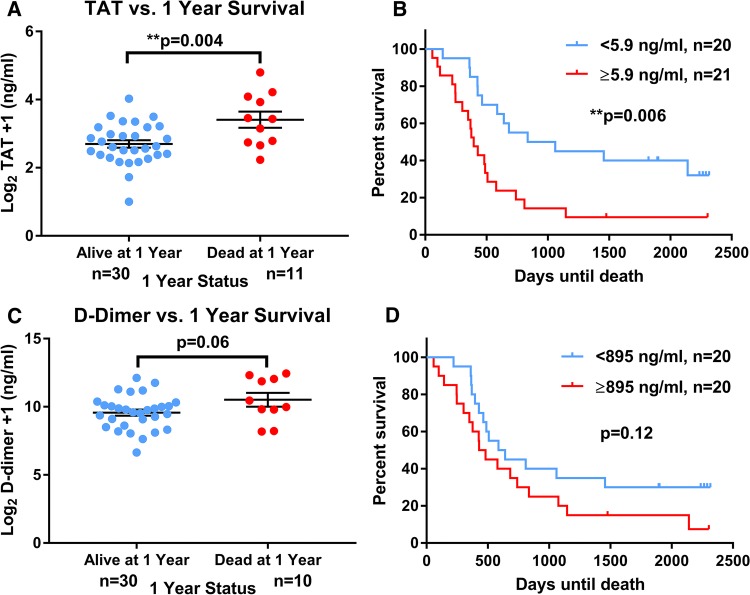
Coagulation plasma markers TAT and d-dimer are associated with poorer survival in metastatic breast cancer. Coagulation markers thrombin–antithrombin III complex (TAT) and d-dimer were quantified by immunoassay in metastatic breast cancer (MBC) patient plasma samples. **a** TAT, **c**d-dimer: unpaired two-tailed *t* tests of Log_2_ transformed raw coagulation marker concentrations compared to survival groups, **p < 0.01. Error bars show standard error of the mean (SEM). **b** TAT, **d**d-dimer: log-rank (Mantel–Cox) tests comparing survival in days from study entry in these coagulation markers dichotomised around the median values (TAT 5.9 mg/ml, d-dimer 895 ng/ml) were carried out, **p < 0.01. Assumption of proportionality was verified based on Schoenfeld residuals; *n* number of MBC patients in each group, *TAT* thrombin–antithrombin III complex

Plasma markers of coagulation did not correlate with ER or Her2 status, or the presence of visceral and locally advanced compared to bone-only metastases. Fibrinogen was not associated with reduced survival, however, plasma TAT was significantly higher in MBC patients dying within 1 year post-recruitment compared to those who survived over a year (median 9.8 vs. 5.2 ng/ml, *p* = 0.004) whilst d-dimer showed a trend for this (median 1211 vs. 817 ng/ml, *p* = 0.06). When dichotomised into high (< 5.9 ng/ml) and low (≥ 5.9 ng/ml), TAT was associated with significantly reduced survival (*p < 0.01, *Fig. 3).

**Fig. 4:**
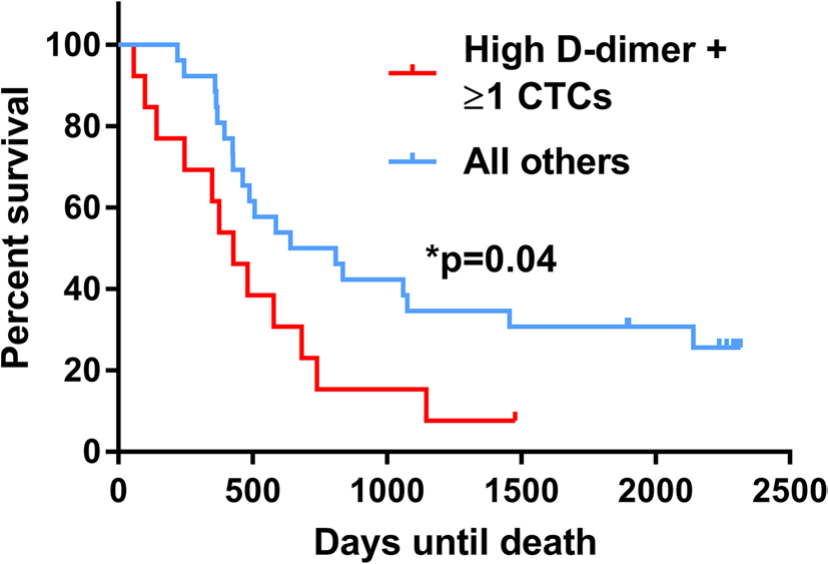
The combined presence of a high d-dimer and circulating tumour cell positivity is associated with reduced survival in metastatic breast cancer. Survival in patients with CTC positive (≥ 1 CTCs/7.5 ml) and high d-dimer (≥ 895 ng/ml) (red line, *n* = 13) was compared to all other patients (blue line, *n* = 26). A log-rank (Mantel–Cox) test comparing survival in days from study entry in the shown CTC/coagulation marker groups was performed. Assumption of proportionality was verified based on Schoenfeld residuals; *n* number of MBC patients in each group, *CTC* circulating tumour cell

MBC patients with a combined high d-dimer (≥ 895 ng/ml) and CTC positive (≥ 1/7.5 ml whole peripheral blood) had significantly reduced survival by log-rank (Mantel–Cox) testing (*p* = 0.04, Fig. 4).

## Discussion

The trend for reduced overall survival in MBC patients with ≥ 1 CTCs/7.5 ml whole blood is consistent with previous reports [30, 31].

The very significant association between plasma TAT and overall survival supports previous work from our team demonstrating higher concentrations of TAT in metastatic compared to early breast cancer patients or age-matched female controls [6]. To our knowledge, here we report the first association between plasma TAT and metastatic breast cancer survival. The trend found between plasma d-dimer and survival supports the findings of Dirix et al. who showed d-dimer to be increased in patients with metastatic compared to early breast cancer or healthy female volunteers and correlates with tumour volume and overall survival [32]. d-dimer has furthermore been reported to be associated with lymphovascular invasion, clinical stage and lymph node involvement in early breast cancer patients, indicating its potential as a prognostic biomarker [10].

The correlation between CTC positivity and plasma coagulation we report here is reflected clinically by the work of Mego et al. [33], where they reported CTC presence was associated with higher plasma d-dimer levels in MBC patients. Both Mego’s findings and or current findings of the association of hypercoagulability and CTCs likely reflects the known symbiotic relationship between circulating tumour cells and the haemostatic system. Tissue factor, the main initiator of the extrinsic pathway of coagulation, assists CTC survival by inhibition of anchorage-dependent apoptosis (anoikis) and through promotion of the epithelial–mesenchymal transition [19, 34, 35]. This was shown to be a two-way relationship by Bourcy et al. who defined a novel induction of tissue factor expression by EMT that drove metastasis in breast cancer. CTCs were shown to jointly express tissue factor and vimentin in MBC patients [36]. Fibrin deposition and platelet activation have been shown to create a ‘thrombus cloak’ that protects circulating CTCs from natural killer cells in murine models [37, 38]. Platelet-derived cytokines have furthermore been demonstrated to block natural killer cell activity, therefore providing a protective effect on CTCs in the bloodstream [39].

Coagulation factors may also help CTCs to extravasate from the vasculature. Platelets help trap CTCs in bloods vessels [40] with ligands expressed by activated platelets aiding adherence to the vascular endothelium [41]. It is postulated that breast cancer CTC expression of tissue factor results in localised fibrin creation, trapping CTCs in platelet–fibrin microemboli and promoting early metastatic seeding into distant organs [36]. Although we did not find any correlation between ER/Her2 status or the site of metastases and CTCs/plasma coagulation, this may simply reflect the small sample size. However, coagulation factor aided-extravasation could provide an explanation for the significantly poorer survival in MBC patients with a combined CTC positivity and high d-dimer, indicating a hypercoagulable microenvironment resulting in increased metastatic potential and worse outcome.

This link between plasma coagulation, CTCs and survival suggests a potential improvement in breast cancer outcome could be achieved by therapeutic targeting of the coagulation system. In vivo breast cancer xenograft models have demonstrated a reduction in tumour growth and metastasis using anti-tissue factor antibodies [42, 43]. The direct thrombin inhibitor dabigatran has also shown efficacy in reducing tumour growth and liver micrometastases in a breast cancer murine model, indicating an anti-cancer effect of the direct oral anticoagulant (DOAC) class of drugs [44]. We are currently studying Rivaroxaban, a DOAC licensed for the prophylaxis and treatment of VTE that is an inhibitor of Factor Xa, thereby inhibiting the TF-Factor VIIa-Factor Xa complex, in a Phase II trial of early breast cancer patients (TIP Trial, EudraCT No.: 2014–004909-33) [45].

In conclusion, this study demonstrates the potential for combining CTC enumeration and assessment of systemic hypercoagulability using TAT or d-dimer as a prognostic biomarker in MBC. In addition, through demonstrating a correlation between hypercoagulability and CTC, this supports the hypothesis that coagulation is mechanistic in promoting CTC intravasation and CTC survival. The coagulation system may, therefore, be a potential therapeutic target in breast cancer.

## Electronic supplementary material

Below is the link to the electronic supplementary material.da

Supplementary file1 (TIF 2731k b)
