# Supplementary figures and images for: Circulating tumour cells and hypercoagulability: a lethal relationship in metastatic breast cancer
Source: Clin Transl Oncol. 2019 Aug 31;22(6):870–7. doi: 10.1007/s12094-019-02197-6 (PMC7188731; doi:10.1007/s12094-019-02197-6)

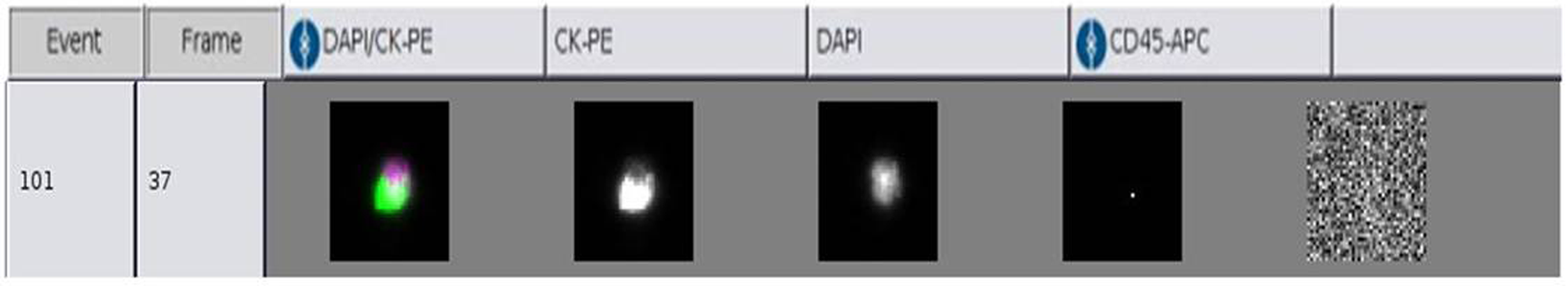

Supplement: Supplementary file 1 — Supplementary file1 (TIF 2731k b) [file 12094_2019_2197_MOESM1_ESM.tif]
